# Liquid biopsy: a step forward towards precision medicine in urologic malignancies

**DOI:** 10.1186/s12943-017-0644-5

**Published:** 2017-04-14

**Authors:** Ashley Di Meo, Jenni Bartlett, Yufeng Cheng, Maria D. Pasic, George M. Yousef

**Affiliations:** 1 Department of Laboratory Medicine and Pathobiology, University of Toronto, Toronto, ON Canada; 2 Department of Laboratory Medicine, Keenan Research Centre for Biomedical Science, Li Ka Shing Knowledge Institute, St. Michael’s Hospital, Toronto, ON Canada; 3 Department of Radiation Oncology, Qilu Hospital of Shandong University, Jinan, Shandong China; 4 Department of Laboratory Medicine, St. Joseph’s Health Centre, Toronto, ON Canada

**Keywords:** Precision medicine, Personalized medicine, Kidney cancer, Bladder cancer, Prostate cancer, Liquid biopsy, Cell-free DNA, Circulating tumor DNA, Circulating tumor cells, Exosomse, Tumor markers, miRNAs, Long non-coding RNA, Biomarkers, Cancer treatment, Predictive markers

## Abstract

There is a growing trend towards exploring the use of a minimally invasive “liquid biopsy” to identify biomarkers in a number of cancers, including urologic malignancies. Multiple aspects can be assessed in circulating cell-free DNA, including cell-free DNA levels, integrity, methylation and mutations. Other prospective liquid biopsy markers include circulating tumor cells, circulating RNAs (miRNA, lncRNAs and mRNAs), cell-free proteins, peptides and exosomes have also emerged as non-invasive cancer biomarkers. These circulating molecules can be detected in various biological fluids, including blood, urine, saliva and seminal plasma. Liquid biopsies hold great promise for personalized medicine due to their ability to provide multiple non-invasive global snapshots of the primary and metastatic tumors. Molecular profiling of circulating molecules has been a stepping-stone to the successful introduction of several non-invasive multi-marker tests into the clinic. In this review, we provide an overview of the current state of cell-free DNA-based kidney, prostate and bladder cancer biomarker research and discuss the potential utility other circulating molecules. We will also discuss the challenges and limitations facing non-invasive cancer biomarker discovery and the benefits of this growing area of translational research.

## Background

The concept of “precision medicine” or individualizing the treatment plan according to the biologic behaviour of the tumor is considered a new epoch in cancer management [1]. The clinical applications of precision medicine are broad, encompassing screening, diagnosis, prognosis, prediction of treatment response and resistance, early detection of recurrence/metastasis and biologic cancer stratification. The goal of precision medicine is to eliminate the “one size fits all” model of patient management, which is centered on average response to care, by shifting the emphasis to tailored treatment according to disease biology and predicted treatment response [2].

**Fig. 1:**
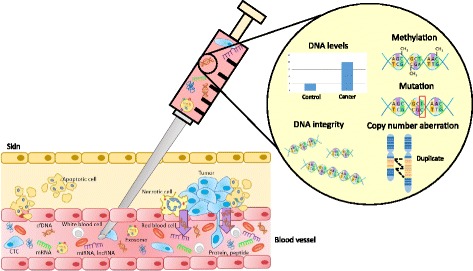
Circulating molecules in liquid biopsy. There are a number of molecules that can be measured in body fluids including cell-free DNA, circulating tumor cells (CTCs), different circulating RNA classes (miRNAs, lncRNAs, mRNAs), cell-free proteins and exosomes. Cell-free DNA escapes into circulation from the primary tumor or metastatic loci through necrosis or apoptosis of tumor cells. Circulating cell-free DNA can then be used as a liquid biopsy to measure DNA levels, integrity, methylation, mutational status and copy number aberration

Liquid biopsy is a non-invasive tool for biomarker discovery that is gaining significant attention. The development of a non-invasive “liquid biopsy” represents a significant innovation in the field of precision medicine. It is capable of replacing, or at least augmenting the use of invasive biopsy which has limited success and associated complications [3, 4]. Liquid biopsies, owing to their minimally invasive nature, are associated with significantly less morbidity and can be scheduled more frequently to provide a personalized snapshot of disease at successive time points. This is particularly valuable during treatment through providing temporal measurements of tumor burden and early evidence of recurrence or resistance [5]. Moreover, liquid biopsy may better reflect the genetic profile of all tumor subclones present in a patient, unlike tissue biopsies which are obtained from only one tumor region [6]. A number of molecules can be isolated from liquid biopsy, as illustrated in Fig. 1. In this review, we provide a detailed discussion on the potential clinical utility of cell-free (cfDNA). We also provide an overview of other circulating molecules, including circulating tumor cells (CTCs), RNAs (miRNAs, lncRNAs, mRNAs), cell-free proteins, peptides and exosomes as cancer biomarkers.

Although circulating cell-free DNA (cfDNA) was first identified in 1948, it has only recently been investigated as a “liquid biopsy” for cancer biomarker detection [7]. Tumors release DNA fragments into circulation that contain tumor-specific alterations including point mutations, copy number variation and DNA methylation (Fig. 1). Although at lower concentrations, cfDNA can also be detected in healthy individuals. Circulating tumor DNA (ctDNA) is highly fragmented, measuring between 180 and 200 base pairs. As a biomarker, ctDNA is easily accessible and reliable. However, ctDNA is rapidly cleared from circulation following surgery or systemic therapy owing to its short half-life, ranging from 16 min to 13 h [8]. Analysis of ctDNA requires highly sensitive techniques. Classic methods for cfDNA assessment include PCR-based approaches. More recently, digital PCR has emerged as a sensitive tool for detection of point mutations in cfDNA. Targeted and whole genome sequencing technologies are also increasingly being applied to cfDNA analysis. Advantages and limitations of a select number of platforms used to assess cfDNA is summarized in Table 1.

**Table 1:** Platforms used to analyze cell-free DNA in circulation

Method	Platform	Applications	Advantages	Limitations	Reference
PCR-based	Nested real-time PCR	• Known point mutations • Methylated genes	• Ease of use • Low cost	• Low sensitivity • Detect limited genomic loci	[129–131]
	Mutant allele-specific PCR				
	Mass spectrometry				
Digital PCR	Droplet digital PCR	• Known point mutations • Methylation	• Very high sensitivity • Quantitative	• Detect limited genomic loci	[132, 133]
	Microfluidic digital PCR				
Targeted Sequencing	Safe-SeqS	• Selected SNV^a^, SCNA^b^ and rearrangements	• High sensitivity • Low cost	• Less comprehensive than next gen sequencing	[134–136]
	Tam-Seq				
	CAPP-Seq				
Whole genome sequencing	karyotyping	• Genome wide SNV, SCNA and rearrangements	• Detect all genomic loci	• Expensive • Time consuming	[137–139]
	PARE^c^				

^a^Single nucleotide variant, ^b^Somatic copy number alteration, ^c^Personalized analysis of rearranged ends

## Advantages and limitations of cell-free DNA

There are several advantages to assessing cfDNA. Sampling is minimally invasive and inexpensive when compared with tissue biopsy [9]. In addition, cfDNA testing can be easily and frequently repeated to monitor changes that occur during treatment, serving as an early indicator of recurrence, resistance, or metastasis [10]. CfDNA most likely reflects the genetic profile of all tumor subclones, unlike tissue biopsy, which does not account for tumor heterogeneity. In patients with renal cell carcinoma (RCC), bladder and prostate cancers, cfDNA is detectable in over 50% of plasma/serum samples and in over 70% of urine samples [11]. Limitations of cfDNA testing include its relatively short half-life. As a result, sampling times are critical. Also, special precautions should be taken for sample reservation. In addition, tumor-specific mutations can represent as low as 0.01% of total cfDNA [9, 12], which can make the detection of rare variants challenging.

## Clinical applications of cell-free DNA

As summarized in Table 2, cfDNA has a broad range of diagnostic, prognostic and predictive applications. By examining several unique characteristics, including circulating cfDNA levels, integrity, methylation and mutational status, researchers have shown that cfDNA has great potential clinical utility for kidney, bladder and prostate cancers.

**Table 2:** Diagnostic, prognostic and predictive applications of cell-free DNA

Clinical application	Cancer type	Sample type	Type of cfDNA analysis	Reference
Predict treatment response	• RCC • Bladder • Prostate	• Plasma • Serum	• cfDNA^a^ level • Methylation • Mutations	[13, 29, 37–40]
Predict recurrence	• RCC • Bladder • Prostate	• Plasma • Urine	• Mutations • Methylation • cfDNA %	[14, 30, 32]
Prognosis	• RCC • Bladder • Prostate	• Plasma • Serum	• ctDNA^b^ level • DNA integrity • Methylation	[20, 23, 35, 36]
Diagnosis	• RCC • Bladder • Prostate	• Urine • Plasma • Serum	• Alteration in DNA level • cfDNA integrity • Methylation • Mutations	[15–18, 20, 21, 24–28] [31, 33, 34]

^a^Cell-free DNA, ^b^Circulating tumor DNA

### Measuring cell-free DNA levels

Several studies have compared cfDNA levels in cancer relative to healthy individuals and those with benign conditions, showing promising diagnostic and prognostic applications.

One study found that plasma cfDNA levels were lower in sorafenib-treated RCC patients with remission relative to those who progressed. Higher cfDNA levels during the course of treatment also indicated poor prognosis [13]. Another study found that plasma cfDNA levels were elevated in metastatic RCC relative to localized disease and could predict postoperative recurrence with 91% sensitivity and 100% specificity [14]. In bladder cancer, urine cfDNA levels were found to be significantly elevated relative to controls [15]. Plasma cfDNA levels were elevated in prostate cancer relative to benign prostatic hyperplasia (BPH) [16], indicating that cfDNA levels can serve as a diagnostic marker for prostate cancer. A consistent study reported that plasma cfDNA levels were higher in prostate cancer patients relative to control subjects with 80% sensitivity and 82% specificity [17].

A similar trend among studies is that cfDNA levels tend to be elevated in cancer patients relative to controls. In addition, a majority of studies report a stepwise increase in cfDNA levels from localized disease to disease progression and metastasis.

### Cell-free DNA integrity

CfDNA is derived from both apoptotic and necrotic cells in cancer patients whereas it predominately originates from apoptotic cells in healthy individuals. cfDNA from apoptotic cells is highly fragmented, whereas DNA from necrotic cancer cells results in longer DNA fragments [18]. CfDNA integrity is a measure of the extent of cfDNA fragmentation and is usually calculated as the ratio of long-to-short cfDNA fragments derived from necrotic and apoptotic cells (necrotic/apoptotic), respectively [19].

CfDNA integrity was found to be elevated in the serum of patients with RCC relative to controls. It was also higher in patients with higher stage (T3) and larger tumor size (>4cm) [20].

A study found that urine cfDNA integrity was elevated in bladder cancer patients relative to healthy individuals and can be used as a marker for early diagnosis [21]. However, reports of decreased cfDNA integrity in bladder cancer patients relative to controls has casted doubts about its diagnostic utility.

cfDNA was also reported to be released by apoptotic and non-apoptotic cell death before and 3 months after prostate cancer diagnosis, whereas it was released only by non-apoptotic cell death 6 months after diagnosis cfDNA [22]. This indicates that cfDNA can be used to follow the evolution of disease especially since repeat liquid biopsy is feasible. Urine cfDNA integrity was elevated in prostate cancer patients relative to healthy individuals with 79% sensitivity and 84% specificity [18]. In a contradictory study, prostate cancer patients were found to have a lower cfDNA integrity in serum relative to BPH patients and healthy controls [23].

Although a majority of studies have observed a cancer-associated elevation in cfDNA integrity suggestive of necrotic cell death, other groups have reported the presence of more fragmented cfDNA and hence lower cfDNA integrity in cancer. The presence of more fragmented cfDNA in bladder and prostate cancer may be a result of cancer-induced apoptosis of peripheral noncancerous tissues. A study found that cfDNA fragmentation displayed a stepwise increase with increasing histological grade [23], again suggesting that high grade tumors may disrupt peripheral tissues resulting in increased apoptosis.

### Cell-free DNA methylation

An important epigenetic change in cancer is methylation changes of tumor-related genes, which can significantly affect the initiation and progression of the disease. Methylation status can be assessed in circulating cfDNA fragments.

An earlier study analyzed methylation of six tumor suppressor genes in urine and concluded that promoter hypermethylation has diagnostic value and is a common and early event in organ-confined kidney cancer [24]. CpG island hypermethylation of serum cfDNA was more frequently observed in patients with RCC relative to controls and was able to diagnose RCC with 63% sensitivity and 87% specificity [25].

Methylation levels of *POU4F2* and *PCDH17* in urine were reported to be able to differentiate bladder cancer from patients with other urological conditions and healthy volunteers with 90% sensitivity and 94% specificity [26]. Methylation status of *TWIST1* and *NID2* in urine could differentiate bladder cancer patients from controls with a combined sensitivity of 90% and combined specificity of 93% [27].

Tumor related genes *RASSF1*, *GSTP1* and *RARB2* were found to be hypermethylated in serum of prostate cancer patients compared to healthy male donors [28]. Plasma level of methylated *GSTP1* DNA was shown to be reduced following chemotherapy [29], indicating that methylated *GSTP1* is a potential predictive marker for chemotherapy response. Elevated plasma cfDNA methylation of *SRD5A2* and *CYP11A1* was seen in prostate cancer patients with biochemical recurrence following radical prostatectomy [30], indicating that aberrant cfDNA methylation can serve as an early predictor for disease recurrence.

Although promising results were obtained from the above studies, it has to be noted that many of these findings still await validation in larger independent cohorts.

### Cell-free DNA mutations

Cancer initiation and progression are triggered by the acquisition of somatic DNA mutations and chromosomal aberrations. The finding that tumor-derived DNA is released into circulation and that mutations in cfDNA can be detected in various biological fluids has prompted investigations into their use as non-invasive cancer biomarkers.

An early study was able to identify chromosome 3p microsatellite alterations in plasma DNA from patients with ccRCC relative to healthy controls [31], indicating potential diagnostic value.

Microsatellite alterations have also been detected in the circulating DNA of bladder cancer patients. Urinary *TERT* promoter mutations were found to correlate with bladder cancer recurrence [32]. *KRAS2* mutation was found to be detectable in plasma ahead of bladder cancer clinical diagnosis [33], indicating that cfDNA mutations can serve as early diagnostic markers.

A panel of chromosomal variations detected in serum could discriminate prostate cancer from controls with a diagnostic accuracy of 83%. This signature was also able to differentiate benign prostatic hypertrophy and prostatitis from prostate cancer with and accuracy of 90% [34]. Focal somatic copy number alteration (SCNA) status was assessed in plasma cfDNA at multiple time points during progression of metastatic prostate cancer. Newly occurring focal amplifications (*AR* and *MYC*) were reported in 40% of patients with metastatic progression, indicating that newly occurring focal amplifications may be useful prognostic biomarkers in a subset of patients [35]. High-level copy number gains in the *AR* locus were detected in the plasma of castration resistant prostate cancer (CRPC) patients but not in castration sensitive prostate cancer (CSPC) patients, suggesting that *AR* copy number gain can serve as a prognostic marker [36]. Plasma androgen receptor (*AR*) mutations were detected in enzalutamide-resistant and abiraterone-resistant patients with metastatic CRPC [37, 38]. Copy number variation of serum *CYP17A1* and *AR* genes was assessed in metastatic CRPC patients who received docetaxel-based chemotherapy followed by abiraterone treatment. The authors found that patients with *AR* and *CYP17A1* copy number gain had shorter progression free survival (PFS) and overall survival (OS) compared to metastatic CRPC patients with no gain. This suggests that *AR* and *CYP17A1* copy number gain may be useful markers for abiraterone resistance [39]. A consistent study found that plasma *AR* copy number gain was associated with abiraterone resistance in metastatic CRPC patients [40].

Although the diagnostic accuracy of specific cfDNA mutations is high, the detection of rare variants can be challenging. This is in part due to the fact that tumor-specific mutations can represent as low as 0.01% of total cfDNA. A recent trend has emerged that looks to assess global chromosomal structural instability instead of individual alterations [34]. This may prove diagnostically useful, especially in patients with rare variants.

## Other circulating molecules and their clinical applications

**Fig. 2:**
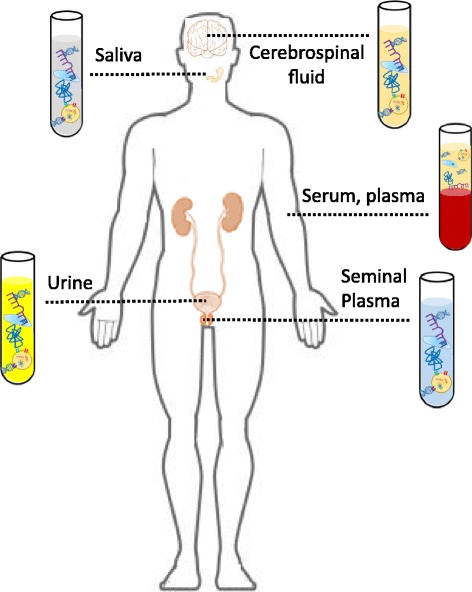
Circulating molecules can be detected in various biological fluids. Circulating molecules are present in a number of biological fluids, including urine, serum, plasma, cerebrospinal fluid, seminal plasma and saliva. These can be obtained using a liquid biopsy

In addition to cell-free DNA, circulating tumor cells (CTCs), circulating RNAs (miRNA, lncRNAs and mRNAs), proteins and peptides as well as exosomes have emerged as a “liquid biopsy” for non-invasive cancer biomarker discovery. Table 3 shows a number of promising diagnostic, prognostic and predictive applications for these molecules. As illustrated in Fig. 2, these molecules can be detected in a number of biological fluids.

**Table 3:** Diagnostic, prognostic and predictive applications of selected circulating molecules

Molecule	Clinical application	Cancer type	Markers	References
CTCs^a^	Diagnostic	Bladder cancer	Cell count	[49]
	Prognostic	RCC, bladder and prostate cancer	Cell count	[45, 51]
	Predictive (recurrence and treatment response)	Prostate cancer	Cell count	[52]
miRNAs	Diagnostic	RCC, bladder and prostate cancer	miR-210, miR-1233, miR-125b, miR-126, let-7e, let-7c, miR-30c, miR-622, and miR-1285	[63]
	Prognostic	Bladder and prostate cancer	miR-146a-5p	[57]
	Predictive (treatment response)	Prostate cancer	miR-21	[68]
lncRNAs^b^	Diagnostic	RCC, bladder and prostate cancer	PCA3, lncRNA-LET, PVT1, PANDAR, PTENP1, linc00963, UCA1, lncRNA H19	[73–75, 77]
	Prognostic	Prostate cancer	PCAT18	[79]
	Predictive (treatment response)	Bladder cancer	UCA1	[76]
mRNAs^c^	Diagnostic	RCC, bladder and prostate cancer	CAIX, UBE2C	[83, 85]
	Prognostic	RCC, bladder and prostate cancer	B7-H3, CK20, cBMP6	[84, 86]
	Predictive (recurrence and treatment response)	Prostate cancer	AR-V7, PSCA,	[89, 90]
Proteins	Diagnostic	RCC, bladder and prostate cancer	AQP1, PLIN2, APOA1, APOA 2, APOB, APOC2, APOC3, APOE, β-MSMB	[99, 100, 104, 105, 108]
	Prognostic	RCC and prostate cancer	Hsp27, KNG1, APOD, FG, HP, CAV1, CAV2	[98, 109]
Peptides	Diagnostic	RCC and prostate cancer	-	[101, 110]
	Prognostic	Bladder cancer	-	[106, 107]
Exosomes	Diagnostic	RCC and prostate cancer	miR-126-3p, miR-449a, miR-34b-5p, miR-34a, miR-148a	[118, 119, 122]
	Prognostic	Bladder cancer	HOTAIR, HOX-AS-2, ANRIL, linc-RoR	[120]
	Predictive (recurrence and treatment response)	RCC and prostate cancer	LncARSR, MDR-1, MDR -3, PABP4	[119, 122, 123]

^a^Circulating tumor cell, ^b^Long non-coding RNA, ^c^Messenger RNA

## Circulating tumor cells

Circulating tumor cells (CTCs) in peripheral blood originate from the primary tumor or metastatic foci. They are estimated to account for at most one cell in a hundred million cells that are circulating in the blood [41]. Thus, identification and characterization of CTCs requires methods with extremely high analytical sensitivity and specificity [42]. Despite their rarity, significant interest has focused on examining the utility of CTCs as cancer biomarkers. The Food and Drug Administration (FDA) has approved the CELLSEARCH^®^ CTC Test for monitoring of patients with metastatic prostate cancer. This test counts CTCs of epithelial origin (*CD45*-, *EPCAM*+, and cytokeratins 8, 18+, and/or 19+) in whole blood [43]. CTC counts can serve as an estimate for disease burden, and changes in CTC counts over the course of systemic therapy can be indicative of treatment response [44].

Blood CTC levels were found to be elevated in RCC patients with advanced stage and were associated with a more aggressive phenotype [45]. Circulating RCC cells were able to predict current and future metastases. Levels of CTCs in peripheral blood were also found to correlate with lymph node status and presence of metastasis in RCC. Enumeration of CTCs in peripheral blood in addition to CTC vimentin expression status was found to be significantly associated with RCC progression [46].

Several groups have assessed the prognostic value of the CELLSEARCH^®^ platform in bladder cancer. Circulating urothelial cancer cells were detected in patients with metastatic bladder cancer by the CELLSEARCH™ assay [47, 48], suggesting that this system can serve as a marker for metastatic bladder cancer. Levels of CTCs were higher in both serum and urine of urothelial carcinoma patients relative to controls. Quantification of CTC based on the high expression of folate receptor α (FRα) had an 82% sensitivity and 62% specificity for bladder cancer detection [49]. CTC enumeration in peripheral blood was also found to be a powerful predictor of early urothelial carcinoma recurrence and cancer-specific and overall mortality [50].

CTCs are detected at high frequency in castration-resistant prostate cancer (CRPC) and are correlated with clinical outcome [51]. In a phase III clinical study, rising CTC levels in patients with metastatic CRPC after three cycles of docetaxel with lenalidomide chemotherapy could predict poor survival [52]. In another phase III trail, whole blood CTC count and LDH level was strongly predictive of overall survival in patients with metastatic CRPC who were previously treated with docetaxel and received abiraterone acetate [53].

CellSearch™ is currently the only CTC assay that has received FDA-approval for prognostic evaluation of prostate, colon, breast and lung cancers in the clinic. Due to problems associated with sensitivity and specificity, CTCs have not yet been fully accepted into clinical practice for guiding treatment decisions. More recently, research groups are moving towards analyzing CTC content (e.g. miRNAs) for detection of cancer biomarkers [54].

## Circulating RNAs

A number of RNA classes, including messenger RNAs (mRNAs), microRNAs (miRNAs) and long non-coding RNAs (lncRNAs) have gained recognition as potential non-invasive cancer biomarkers [55]. Several reports found altered levels of circulating RNAs in cancer, which returned to normal following surgery [56, 57], suggesting a tumor-associated release of RNA molecules.

### miRNAs

miRNAs are short non-coding RNAs, 21–23 nucleotides in length, that regulate gene expression by pairing to the 3′untranslated region (UTR) of their target mRNA [58]. The link between miRNAs and caner has been well established in literature as they have been shown to play a key role in tumorigenesis, tumor progression and metastasis [59]. Circulating miRNAs are present in many biological fluids including blood, urine, saliva, tears, and cerebrospinal fluid [60, 61], suggesting that they can be used as non-invasive cancer biomarkers.

Several studies reported elevated serum levels of miR-210 in RCC relative to controls [62, 63]. Circulating levels of miR-221 and miR-222 in plasma were reported to distinguish RCC patients from controls. Moreover, plasma miR-221 levels also presented at higher levels in RCC patients with metastasis [64]. Elevated urine miR-15a levels have been detected in RCC patients and were nearly undetectable in oncocytoma [65].

Consistent reports found urinary levels of miR-126 to be elevated in urothelial carcinoma relative to healthy controls [66]. Urine miR-146a-5p was significantly higher in bladder cancer and was associated with tumor grade and depth of invasion [57].

One study identified four miRNAs that were down regulated and six miRNAs that were upregulated in the sera of prostate cancer patients [67]. Another group found that serum miR-21 was elevated in hormone-refractory prostate cancer (HRPC) patients, especially in those resistant to docetaxel-based chemotherapy [68].

Overall, miRNAs have shown great promise as cancer biomarkers. multiple consistent reports have identified circulating miR-210 as a diagnostic marker in RCC, miR-126 as a diagnostic marker in bladder cancer and miR-21 as prognostic markers for prostate cancer. Although these studies support the use of circulating miRNAs as biomarkers, they have yet to be clinically validated. We speculate that a miRNA signature could overcome this by reducing false-positive and false-negative results.

### Long non-coding RNAs

LncRNAs are > 200 nucleotides in length and can regulate gene expression at the transcriptional, post-transcriptional or epigenetic levels [69]. Accumulating evidence shows that lncRNAs are altered in cancer and can promote tumor formation, progression and metastasis [70]. The application of lncRNAs as non-invasive cancer biomarkers has recently grown interest [71]. Prostate cancer antigen 3 (PCA3) is the most notable example since it is a specific urine marker for prostate cancer. PCA3 is developed into an FDA-approved non-invasive urine test, PROGENSA PCA3, to aid in the decision for repeat biopsy in men 50 years of age or older who have had one or more previous negative prostate biopsies [72, 73].

A panel of five circulating serum lncRNAs (lncRNA-LET, PVT1, PANDAR, PTENP1 and linc00963) was able to differentiate benign renal tumors from ccRCC [74]. Circulating UCA1 levels in urinary sediments were identified as a potential diagnostic marker for urothelial carcinoma with 81% sensitivity and 92% specificity [75]. Blood UCA1 levels were elevated in patients with advanced bladder cancer after cisplatin-based combination chemotherapy [76]. Taken together, circulating UCA1 is a promising biomarker for bladder cancer diagnosis and therapeutic monitoring.

Hypermethylation of the lncRNA H19 in peripheral blood could distinguish prostate cancer from controls [77]. Plasma MALAT1 levels were elevated in prostate cancer. Assessment of MALAT-1 urine levels could prevent approximately 30–46% of unnecessary biopsies in patients with serum PSA level of 4–10 ng/mL [78]. Plasma PCAT18 levels were shown to increase incrementally from healthy individuals to those with localized and metastatic prostate cancer [79].

Overall, although recently discovered, circulating lncRNAs are a promising new class of non-invasive cancer biomarkers that have successfully entered into clinical diagnostics. This may be due to their tissue- and cancer-specific expression patterns. The highly specific expression of lncRNAs may also explain why independent reports support the application of cell-free UCA1 and MALAT1 as diagnostic biomarkers for bladder and prostate cancers, respectively. However, it is clear that a more in-depth understanding of their biology is still required.

### Messenger RNAs

Circulating mRNAs were first reported in cancer patients in the 1990s [80]. Although the vast majority of circulating mRNAs are degraded by RNases [81], some appear to be relatively stable in circulation [82], which is likely a result of complexing with proteins and/or lipid carriers. Due to their role in intracellular protein translation, circulating mRNAs likely reflect the status of intracellular processes and are potential cancer biomarkers.

Percentage of urine CAIX splice variant mRNA was reported to have high diagnostic performance for kidney, prostate and bladder cancers (90% sensitivity and 72% specificity) [83]. Levels of B7-H3 mRNA in peripheral blood were significantly elevated in metastatic RCC [84].

Urine *UBE2C* mRNA levels were significantly higher in bladder cancer patients relative to normal and hematuria samples [85]. Urinary CK20 mRNA was found to be a potential diagnostic marker for urothelial carcinoma. In addition, CK20 was found to increase gradually with tumor grade and stage [86]. Urine *hTERT* mRNA is another potential marker for the early diagnosis and prognosis of bladder cancer [87].

In addition to bladder cancer, *hTERT* mRNA was identified as a useful diagnostic biomarker in prostate cancer and has been linked to poor prognosis [88]. Whole blood AR-V7 levels were associated with response to abiraterone treatment in metastatic CRPC [89], indicating that they can serve as predictive markers. Consistent with this, other groups found that circulating AR-V7 detection could help guide treatment selection in castration resistant prostate cancer [90].

Despite the long history of circulating mRNA discovery, this field has not translated into the clinic, perhaps due to their lack of stability and inter-individual variability of mRNAs in circulation [91]. However, some circulating mRNAs remain promising biomarkers since overlapping results demonstrate the potential utility of AR-V7 in prostate cancer and *hTERT* mRNA in bladder and prostate cancers. The answer here might be to combine circulating molecules into one multi-marker test to improve the accuracy of individuals circulating molecules.

## Circulating proteins and peptides

Proteomic and peptidomic analyses have marked a new horizon for non-invasive cancer biomarker discovery [92, 93]. A number of non-invasive multi-marker tests are commercially available. The Prostate Health Index (PHI) is a blood based test that combines total PSA, free PSA, and [-2] proPSA for prostate cancer detection [94]. The 4KScore is a multi-marker blood test that combines measurement of four kallikreins including total PSA, free PSA, intact PSA, and *hKLK2* for assessment of significant (Gleason > 7) prostate cancer before biopsy [95]. ImmunoCyt™ is urine-based test that detects cytoplasmic mucins and high-molecular-weight carcinoembryonic antigen for urothelial carcinoma diagnosis [96]. The Aura Tek FDP Test™ measures fibrin degradation products (FDPs) in the urine and can detect bladder cancer recurrence [97].

In RCC, elevated serum *Hsp27* was found to be associated with high grade [98]. Urine *AQP1* and *PLIN2* levels were able to distinguish clear cell from papillary RCC with 95% sensitivity and 91% specificity [99]. In a clinical trial, urine *AQP1* and *PLIN2* were identified as screening biomarkers for clear cell and papillary RCC [100]. A panel of 40 urinary peptides were able to discriminate RCC patients from controls with high sensitivity and specificity [101]. Another panel of four serum peptides was found to have 100% sensitivity and 93.3% specificity for RCC diagnosis [102]. A 12 urine peptide signature was reported to differentiate malignant from benign renal masses and controls [103].

Circulating urine levels of *APOA1*, *APOA2*, *APOB*, *APOC2*, *APOC3*, and *APOE* were elevated in bladder cancer relative to healthy controls [104]. Consistently, Chen et al. identified elevated levels of *APOA1* and *APOA2* in the urine of bladder cancer patients with diagnostic potential [105]. A signature of eight urinary peptides derived from abundant serum proteins were found to distinguish patient with non-muscle and muscle-invasive urothelial carcinoma [106], indicating that circulating peptides can serve as a marker for disease progression. Another group identified a signature of four urinary peptides that could distinguish muscle invasive from non-muscle invasive bladder cancer [107].

Urinary *β-MSMB* was lower in patients with prostate cancer relative to benign prostatic conditions. When combined with serum PSA, the sensitivity of prostate cancer detection increased [108]. Plasma *CAV1* and *CAV2* levels were higher in patients with CRPC relative to those with non-castration resistant [109]. A signature of 12 urinary peptides could differentiate prostate cancer from benign prostatic conditions. In combination with age, free and total PSA, this signature had improved detection [110].

It is clear that despite remaining challenges, proteomics shows clinical promise. Several independent studies support the utility of circulating *AQ1* and *APOA1*/*APOA2* as non-invasive markers for RCC and bladder cancer, respectively. Moreover, the improved sensitivity, specificity and the clinical success of multi-marker assays has driven the shift from a single- to multi-marker view. The development of an in-depth reference proteome may also help identify candidate biomarkers that are likely to be translated into the clinic [111, 112].

## Exosomes

Exosomes are actively secreted membrane vesicles, 30–100 nm in size that are present in nearly all body fluids [113]. Exosomes play a key role in intercellular communication through transfer of biologically active molecules and can influence therapeutic response [114]. They are stable carriers of various molecules (RNAs, DNA and proteins) [115] and are present at elevated levels in cancer patients relative to healthy subjects [116]. As such, there is increasing interest in the application of exosomes as non-invasive cancer biomarkers. ExoDx™ *Prostate (IntelliScore)*, is a recently developed FDA-approved non-invasive urine test that assesses the expression of three exosomal RNAs associated with high-grade prostate cancer. The test is used alongside PSA to distinguish high grade (Gleason score ≥ GS7) from low grade cancers [117].

A recent study reported that exosomal miRNAs could differentiate benign lesions from ccRCC and healthy individuals [118]. Recent published data indicates that exosomal lncRNAs have potential to serve as predictive biomarkers for guiding treatment. Exosomal lncARSR in plasma was elevated in RCC patients and could predict poor response to sunitinib. Moreover, LncARSR levels were found to decrease following surgical resection and increase at relapse [119].

The lnRNAs HOTAIR, HOX-AS-2, ANRIL and linc-RoR were enriched in urinary exosomes from urothelial cancer patients with high-grade muscle-invasive disease [120]. The levels of twenty-four proteins isolated from urinary exosomes were significantly altered in bladder cancer. The study also showed a strong association between exosomal levels of *TACSTD2* and bladder cancer [121].

Exosomal levels of miR-34a, miR-148a were significantly reduced in the urine of prostate cancer relative to BPH [122]. Exosomal serum *MDR-1*, *MDR-3* and *PABP4* proteins were enriched in docetaxel-resistant CRPC patients relative to doxetaxel-sensitive patients [123]. Another study found that serum exosomal P-glycoprotein were higher in docetaxel-resistant patients than therapy-naïve patients [124], indicating that exosomal P-glycoprotein may be a potential marker for docetaxel resistance.

Although a promising source of cancer biomarkers, few exosomal biomarkers have been implemented into clinical practice. This is partly due to the lack of accurate isolation and detection methods. We speculate that the development of sensitive capture platforms is likely to trigger the introduction of novel exosomal biomarkers into the clinic in the near future.

## Challenges facing liquid biopsy

It is clear that liquid biopsies are a promising revolution in the field of biomarker research. Although there is great potential to influence patient care, there are a number of biological, technical and clinical challenges that need to be addressed before liquid biomarkers are adopted into clinical practice. In theory, circulating molecules should reflect the tumor. However, not all tumor loci are identical. As such, a key challenge is understanding where these molecules are coming from, whether they arise from the primary tumor or metastatic lesions. There is also a need for an incredible amount of assay sensitivity since these molecules are present at low levels in biological fluids. Although this can be achieved using next generation sequencing platforms and droplet digital PCR (ddPCR), the amount of material collected needs to be sufficient for analysis. Another challenge is determining whether the amount of circulating molecules in a biological fluid is sufficient to detect minute alterations. If not, rare alterations would be missed. Assessment of global alterations, such as chromosomal structural instability may help to overcome this. The biological fluid selected for study should also be relevant to the clinical question of interest. Some biological fluids are more complex than others (blood being more complex than urine) making analysis more challenging.

## Future perspective

Circulating cancer biomarker development is a rapidly growing field. Recent evidence suggests a real clinical value. Table 4 provides a partial list of commercially available tests. Although the concept of non-invasive biomarker detection is not new, recent enthusiasm is triggered by advances in technology. It is not surprising that the introduction of next generation sequencing and ddPCR have allowed for improved detection and reduced operating costs and time. The recent introduction of specialized collection tubes has also contributed to this by allowing clinical laboratories to preserve and stabilize circulating molecules in blood and plasma. Although there are examples of FDA-approved circulating markers in the clinic, the majority of markers are still experimental. In a recent study, it was clear that a “biomarker panel” had improved sensitivity and specificity compared to single markers [125]. We speculate that the shift from a single- to multi-marker view will be instrumental in pushing the field forward. In future, it may also be beneficial to combine different levels of molecular alterations (combining genomic, transcriptomic and proteomic) to improve diagnostic, prognostic and predictive accuracy [126, 127]. To ensure that the path from discovery to clinical diagnostics continues to be successfully paved, the analytic, diagnostic and regulatory requirements of a clinical assay need to be understood. Furthermore, active partnerships with industry and effective communication between clinicians and scientists are also necessary.

**Table 4:** Partial list of commercially available circulating tumor markers for urologic malignancies

Test name	Molecules assessed	Cancer type	Clinical application	Biological fluid tested	Reference
CELLSEARCH^®^ CTC Test	CTC^a^	Prostate	Prognostic for patients with metastatic prostate cancer	Blood	[44]
PROGENSA PCA3 Test	lncRNA PCA3	Prostate	Diagnostic for prostate cancer patients with previous negative biopsy (Determine need for repeat biopsy)	Urine	[73]
Prostate Health Index (PHI)	Protein (total PSA, free PSA, and [-2] proPSA)	Prostate	Diagnostic for prostate cancer patients with a PSA between 4 and 10 ng/mL	Blood	[94]
4KScore	Protein (total PSA, free PSA, intact PSA, and human KLK 2)	Prostate	Prognostic (Assess risk for aggressive prostate cancer)	Blood	[95]
ImmunoCyt™ Test	Protein (mucins and HMW carcinoembryonic antigen)	Bladder	Diagnostic for G1, G2 and G3 bladder cancer patients with positive urine cytology	Urine	[96]
Aura Tek FDP Test™	Protein (fibrin degradation product)	Bladder	Predictive of bladder cancer recurrence	Urine	[97]
ExoDx*™* Prostate (IntelliScore)	Exosomal RNA	Prostate	Prognostic for high-grade prostate cancer at the time of biopsy and at surgery	Urine	[117]

^a^Circulating tumor cell

## Conclusion﻿s

It is clear that we are moving into an era of precision medicine, where treatment is tailored based on tumor behaviour rather than the average response to therapy [128]. Molecular profiling, the global analysis of genomic, transcriptomic and/or proteomic profiles, represents a critical pre-requisite for the successful development of individualized treatment strategies. Liquid biopsy is a promising non-invasive tool for molecular profiling, enabling assessment of cfDNA and other circulating molecules in various biological fluids for biomarker discovery. So far, the most exciting applications of liquid biopsies seem to be prognosis and early assessment of treatment failure.

## Key points

- Molecular profiling is becoming the basis for “precision medicine” or individualized treatment.
- Liquid biopsy is a non-invasive tool that can provide a global snapshot of the primary and metastatic tumors.
- Circulating cell-free DNA levels, integrity, methylation and mutational status have promising clinical applications in the field of urological cancer biomarker discovery.
- Circulating tumor cells, circulating RNAs (miRNA, lncRNA and mRNA), cell-free proteins and exosomes obtained through liquid biopsy are promising biomarkers and can provide additional insight into tumor biology.
- A key challenge facing liquid biopsy is understanding where these circulating molecules are coming from, whether they arise from the primary tumor or from the metastatic lesion.
- The shift from a single- to multi-marker view is likely to ensure that the path from discovery to clinical diagnostics continues to be successfully paved.

## Abbreviations

BPH: Benign prostate hyperplasia
ccRCC: clear cell renal cell carcinoma
cfDNA: Cell-free DNA
CRPC: Castration-resistant prostate cancer
CTC: Circulating tumor cell
ctDNA: circulating tumor DNA
HRPC: Hormone-refractory prostate cancer
lncRNA: long non-coding RNA
SCNA: Somatic copy number aberration
